# Use of Gnawing Hay Blocks: Effects on Productive Performance, Behavior and Reactivity of Growing Rabbits Kept in Parks with Different Sex-Group Compositions

**DOI:** 10.3390/ani12091212

**Published:** 2022-05-08

**Authors:** Marco Birolo, Angela Trocino, Andrea Zuffellato, Fabrizio Pirrone, Francesco Bordignon, Gerolamo Xiccato

**Affiliations:** 1 Department of Agronomy, Food, Natural Resources, Animal and Environment (DAFNAE), University of Padova, Viale dell’Università 16, 35020 Padova, Italy; marco.birolo@unipd.it (M.B.); francesco.bordignon@unipd.it (F.B.); gerolamo.xiccato@unipd.it (G.X.); 2 Department of Comparative Biomedicine and Food Science (BCA), University of Padova, Viale dell’Università 16, 35020 Padova, Italy; fabrizio.pirrone@phd.unipd.it; 3 A.I.A. Agricola Italiana Alimentare S.p.A., Piazzale Apollinare Veronesi, 37036 Verona, Italy; andrea.zuffellato@aia-spa.it

**Keywords:** group housing, budget time, aggression, open-field test, novel-object test, human approach test

## Abstract

**Simple Summary:**

Alternative cage-free housing systems for growing rabbits, such as parks/elevated pens, are currently being developed in Mediterranean countries. In these parks, the inability to express gnawing behavior and fear are the top behavioral-welfare-associated consequences. Gnawing inability promotes abnormal stereotypical and aggressive behaviors. Fear can be reduced by avoiding aggression. Thus, the present study aims to evaluate the effect of gnawing compressed hay blocks and sex-group composition (only females, only males, mixed sex) on the productive results, behavior, and reactivity of growing rabbits in a park system. None of the animals showed injuries due to aggression at the end of the trial. The provision of gnawing blocks affected rabbit behavior negligibly but improved their reactivity towards a new environment or a new object; thus, the inclusion of gnawing blocks should be recommended for animal welfare. Changes in sex-group composition were not relevant in terms of either quantity or quality of performance and conspecific interactions.

**Abstract:**

To improve animal welfare in collective park housing systems, this study assessed the effects of the provision of gnawing hay blocks and the group composition (F: females, M: males, FM: mixed sex) on performance, behavior, and reactivity of 288 growing rabbits reared in 18 parks (16 rabbits/park) from 31 to 73 days of age. The presence of gnawing blocks inside the feeding area of the parks scarcely affected performance and budget time, but decreased the time spent in the resting area compared to parks without blocks (*p* < 0.001); it increased the time spent moving during the open field test (*p* < 0.05) and the rate of rabbits that approached the object in the novel-object test (*p* < 0.05). As for sex-group composition, the feed conversion ratio was lower in parks F and FM than in park M (*p* < 0.05). During the open-field test, FM rabbits spent more time moving (*p* < 0.05), whereas M rabbits displayed self-grooming for a longer time (*p* < 0.01). Results related to production, behavior, and reactivity indicate the provision of gnawing blocks for welfare improvement, but do not support the change from current mixed-sex to single-sex rearing.

## 1. Introduction

In the European Union (EU), meat-rabbit farming is concentrated in three member states: Spain, France, and Italy (about 85% of EU production) [1,2], which still rear growing rabbits in small conventional barren bicellular or dual-purpose cages (with 2 or 4–6 rabbits, respectively), and more recently, enriched cages; that is, larger and higher cages equipped with platforms [1,2]. Nevertheless, the European citizens’ initiative “End the Cage Age” asks for the banning of any cage system for farmed animals in the EU, including rabbits, for which the European Parliament is calling for the phasing out of cages by 2027 [3].

Initially used in Belgium and the Netherlands, alternative housing systems such as parks (also called elevated pens) are at a developmental stage in countries that are the main producers of meat rabbits and require improvements and standardization in terms of management and equipment [1]. They usually consist of open-top parks, where growing rabbits can be reared in groups of different sizes (more than 8, litter size; often 30–32). Generally equipped with plastic flooring or wire-net floors covered by plastic footrest pads, parks often include elevated platforms [1].

In these systems (elevated pens/parks), according to EFSA [1], the welfare of growing rabbits is “likely/highly likely to be higher”, whereas it is lower in conventional bicellular and dual-purpose cages. In parks, among the five top main welfare consequences (i.e., problems) (WC) [1], besides two health-related WCs (i.e., skin and gastrointestinal disorders), three behavior-related ones (i.e., resting problems, inability to express gnawing behavior, and fear) need to be addressed for the successful implementation of park systems and a further improvement of animal welfare in these systems.

Resting problems can be related to stocking density, use of appropriate flooring materials, and good hygiene. Gnawing inability is known to promote abnormal stereotypic and aggressive behaviors based on studies performed under different housing conditions [4,5,6,7,8,9]. However, there is a lack of consistency regarding the effects of the presence of gnawing materials on other rabbits’ behaviors, as well as a lack of evidence regarding the material that can best satisfy the rabbit’s gnawing motivation [1]. Finally, fear can be reduced by avoiding rough handling and situations that contribute to aggression [1]. These latter situations are more likely to occur under the conditions of collective housing in a park system, whereas the occurrence of aggressive behaviors and skin wounds has been correlated with an increase in group size [1,10,11]. In this regard, whether group composition in terms of sex can affect aggression among animals is not clearly stated, since few contradictory results are available [9,12,13,14], whereas rabbits are usually kept in mixed-sex groups rather than in single-sex groups.

Thus, the present study aims to evaluate the effect of the presence of gnawing materials (as compressed hay blocks) and sex-group composition (only females, only males, females and males) on the productive results, behavior, and reactivity of growing rabbits kept in a park system.

## 2. Materials and Methods

### 2.1. Facilities, Animals, and Experimental Conditions

The trial ran on the rabbit farm of the University of Padova in a closed building from October to November under a natural photoperiod (approximately 11–12 h of lightness). Extraction fans and an automatic heating system guaranteed air quality and maintained the temperature between 20 °C and 24 °C.

At 31 days of age, 288 crossbred rabbits (144 females and 144 males; Hypharm, Groupe Grimaud, Roussay, Sevremoine, France) were selected from healthy litters of multiparous does (≥3 kindling) in a commercial farm, transported to the experimental farm, individually identified using ear marks and housed in 18 parks (1.28 m front/back walls × 0.78 m side walls; area: 1 m^2^) with 16 animals per park (16 animals/m^2^), i.e., the typical stocking density used in commercial farms [13]. The back/front walls and the side walls of the parks (1.10 m-height) were made of galvanized wire nets (grids: 20 mm × 20 mm; diameter: 2 mm). The parks were composed by two modules of the same dimensions (0.64 m × 0.78 m) partially separated by a wire-net wall (0.35 m × 1.10 m-height) between them, leaving a 0.43 m passage between the two modules. The first module represented the feeding area (0.5 m^2^; left side of the park), and the second module was the resting area (0.5 m^2^, right side of the park). The feeding area had a wire-net floor (grids: 75 mm × 15 mm, diameter: 2.5 mm), four automatic nipple drinkers, and a feeder (0.60 m-width) fixed at the front wall. The resting area had a plastic slatted floor with rectangular holes (70 mm-length × 10 mm-width; distance between the holes: 7 mm) and was equipped with two additional nipple drinkers.

The study was designed as a bifactorial arrangement with three sex-group compositions per park (F: 16 females/park; M: 16 males/park; FM: 8 females and 8 males/park), combined with the absence or presence of gnawing hay blocks (replicates: 3 parks per experimental group). Hay blocks (cylinder form; length: 400 mm; diameter: 80 mm; dry matter: 89.0%; crude protein: 11.2%; ether extract: 1.4%; neutral-detergent fiber: 53.1%; acid-detergent fiber: 34.7%; acid-detergent lignin: 9.1%) were provided ad libitum during the whole trial in a metal tube (400 mm length, diameter: 100 mm) fixed at the back wall of the park feeding area.

All rabbits were fed ad libitum, had free access to fresh water, and received a post-weaning diet (dry matter: 88.8%, crude protein: 15.4%; ether extract: 4.1%; neutral-detergent fiber: 37.1%; acid-detergent fiber: 19.6%; acid-detergent lignin: 5.1%) from 31 to 55 days of age, followed by a fattening diet (dry matter: 88.5%, crude protein: 15.5%; ether extract: 4.4%; neutral-detergent fiber: 35.0%; acid-detergent fiber: 18.0%; acid-detergent lignin: 4.1%) from 56 days of age until slaughter (73 days). The diets were formulated to meet the nutritional requirements of growing rabbits according to de Blas and Mateos [15] and did not contain any antibiotics or coccidiostat.

### 2.2. Growth Performance and Health

During the trial, the individual live weight of the animals and hay-block consumption (where present) of the park were recorded once a week, and park-feed consumption was recorded daily. Hay-block consumption was measured as the difference between initials and final weights of blocks. The morbidity and mortality rates were monitored daily. Rabbits were considered ill when they showed diarrhea and/or mucus in the feces or live weight loss during a week. The day before slaughter, the presence and severity of body injuries caused by aggression were assessed.

### 2.3. Behavioral Recordings and Observations

At 42 and 70 days of age (i.e., around half of the trial and towards the end of the trial), the behavior of the rabbits was video-recorded using infrared cameras for 24 h. Then, over two minutes per hour in each park for 24 h at the two ages, the following behaviors were recorded and expressed as a percentage of the total observation time: time spent feeding, drinking, cecotrophy, resting (crouched body, with the abdomen in contact with the floor; stretched body, with both fore and hind legs stretched beside the abdomen in contact with the floor), self-grooming, allo-grooming, moving, running, sitting, biting/licking, and sniffing (16). Time spent in the feeding and resting areas of the pens was also measured. Finally, the occurrence of rearing, hops, aggressive interactions, and stereotypic behaviors was recorded and expressed as the number of events per park per observation interval [16].

### 2.4. Open-Field Test

At 64 days of age, 108 rabbits (6 animals per park; 3 females and 3 males in the case of FM parks) were subjected to an open-field test to evaluate their reactivity towards a new environment [17,18]. The rabbits to be tested were randomly taken out of cages. In the case of FM parks, the sex of the rabbits was immediately checked based on the individual animal identification. The test was conducted in an arena (2 m × 2 m) with 0.80-m-high wooden walls and a plastic floor divided into nine numbered squares, placed in a contiguous room in the same stable where rabbits were kept. The total duration of each test was 10 min per animal. Each rabbit was placed in a closed wooden box (0.22 m-length × 0.30 m-width × 0.30 m-high) connected to the arena by a sliding door. After one minute, the sliding door was opened, the number (n) of attempts made by the rabbit, and the time (latency, s) spent to enter the arena was recorded for another minute. If, after this minute, the rabbit was still in the box, it was gently pushed into the arena, the sliding door was closed, and the behavior of the rabbit was video-recorded for 8 min. The behaviors evaluated during the open-field test were total displacement (n), central displacement (n), movement (s), running (s), exploration (s), escape attempts (n), hops (n), standing still (s), rearing (n), grooming (s), digging (s), biting (s), resting (s), defecation (n), and urination (n) [17,18].

### 2.5. Novel-Object Test

At 65 days of age, reactivity towards a new stimulus was evaluated using a novel-object test in all parks [19]. The novel object was a 1.5 L plastic bottle, half full of water, anchored by a cap with an iron chain, and dropped from the roof in the center of each park a few centimeters above the floor. The behavior in each park was video-recorded for 5 min, and the number of animals that touched the object was measured during the first minute (1–60 s), during the following two minutes (61–180 s), and the last two minutes (181–300 s), and then expressed as a percentage of the total number of rabbits in the park.

### 2.6. Human-Approach Test

At 67 days of age, the human-approach test was used to measure the animal fear level towards men in all parks [18,19,20]. An unfamiliar operator opened each park and placed a hand at the center of the park a few centimeters above the floor (at the animals’ withers’ height). Rabbit behavior was video-recorded for 5 min. The number of rabbits that touched or sniffed the operator was measured during the first minute (1–60 s), during the following two minutes (61–180 s), and the last two minutes (181–300 s) and expressed as a percentage of the total number of rabbits in the park.

### 2.7. Commercial Slaughtering

At 73 days of age, after a 4 h fasting period, the rabbits were weighed at the experimental farm. The rabbits that reached the minimum live weight requested by the slaughterhouse (2.3 kg) were caged and transported to a commercial slaughterhouse by an authorized truck, which took approximately 1 h of transport. Slaughtering took place approximately 1 h after their arrival at the slaughterhouse, where the rabbits were individually weighed, stunned by electroanesthesia, and killed by jugulation. After 2.5 h chilling, commercial carcasses were weighed to calculate the individual slaughter yield [21].

### 2.8. Statistical Analysis

Individual live weight, daily weight gain, and slaughter yield data were subjected to ANOVA using the PROC MIXED of SAS (SAS 9.4 software, SAS Institute Inc., Cary, NC, USA) with gnawing blocks (absence vs. presence) and sex-group composition (F vs. M vs. FM) as the main effects with interaction, and the park as a random effect. Park-feed intake and feed conversion were analyzed using PROC GLM, considering the same main effects.

The time spent on the different behaviors and the rate of rabbits in the feeding or resting areas of the parks were analyzed by a generalized linear mixed model using PROC GLIMMIX with gnawing blocks, sex-group composition, and rabbit age as the main effects with interactions. The observation time was a random effect, and data from the same park were considered as repeated measures. An underlying Poisson distribution was assumed for all the data.

Data from the reactivity tests (open-field, novel-object, human-approach) were analyzed using the PROC GLIMMIX with a generalized linear mixed model considering gnawing blocks and group composition as the main effects with interaction, and park as a random effect. An underlying Poisson distribution was assumed for the data. The percentage of animals entering the arena spontaneously in the open-field test was coded as a binary variable (entering = Yes or No) and evaluated by maximum-likelihood analysis using the CATMOD procedure, which used gnawing blocks, group composition, animal age as the main effects with interactions.

The means were compared using Bonferroni’s test. Differences among means with a *p*-value < 0.05 were accepted as representing statistically significant differences.

## 3. Results

### 3.1. Rabbit Health and Production Results

During the trial, five rabbits died because of enteric disorders, and the other seven were discarded at the end of the trial because of their low live weight (<2.3 kg). In details, in the parks without gnawing blocks, two rabbits of F parks and two rabbits of M parks died or were discarded; in parks with the gnawing blocks, three, one, and four rabbits died or were discarded from F parks, M parks, and FM parks, respectively. Thus, the mortality rate was 1.7% and total losses (death + discarded animals) were 4.2%, without significant differences among the experimental groups (data not reported in tables). Neither aggressive behavior nor the presence of body lesions were observed during the trial.

The presence of gnawing blocks affected rabbit performance by increasing daily weight gain from 31 to 52 days of age (+2.0 g/day; *p* < 0.05) and throughout the whole trial (+1.2 g/day; *p* < 0.05), which was associated with a higher weight at slaughter (73 days) (+55 g; *p* < 0.05) (Table 1). Feed intake and conversion were not significantly affected. The consumption of compressed hay blocks was 1.3 g/day per rabbit during the whole trial, regardless of sex-group composition (data not reported in tables).

As for sex-group composition, parks with only females and with both sexes had lower feed-conversion ratios compared to parks with only males (*p* < 0.05). Males also had a higher slaughter yield than females (+0.9 percentage points; *p* < 0.01), whereas intermediate values were recorded in mixed-sex parks.

### 3.2. Behavioral Observations

The presence of gnawing blocks did not affect the budget time of growing rabbits (Table 2), except for the time spent allo-grooming, which was higher in parks without blocks than in those with blocks (0.75% vs. 0.44% observation time; *p* < 0.001). In addition, the time spent in the resting area was higher in parks without blocks (53.5% vs. 49.4%; *p* < 0.001) (Table 2). Regarding the effect of group composition, rabbits in mixed-sex parks spent more time resting than those in single-sex parks (67.8% observation time in FM vs. 64.2% and 64.7% in F and M parks, respectively; *p* < 0.01). The time spent in self-grooming was lower in the FM parks than in the F parks and M parks (12.8% vs. 15.4% and 15.8% observation time; *p* < 0.001), whereas the opposite trend was observed for the time spent in allo-grooming (0.73% in FM parks vs. 0.60% and 0.46% in F and M parks, respectively; *p* < 0.01). Stretching was observed more often in rabbits from FM parks than in those from F and M parks (+0.08 events per park; *p* < 0.05). Finally, the time spent in the resting area was higher in parks with only females than in those with both sexes (+2.3 percentage points; *p* < 0.05) (Table 2).

**Figure 1:**
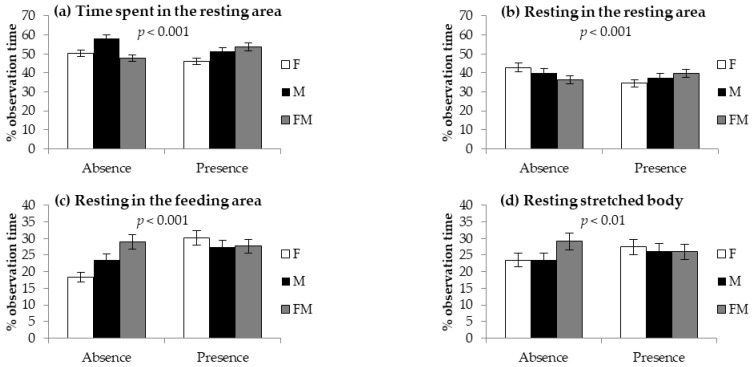
Time (% of observation time) spent (**a**) in the resting area, (**b**) resting in the resting area, (**c**) resting in the feeding area, and (**d**) resting with stretched body in growing rabbits kept in collective parks with only females (F), with only males (M), or with females and males (FM) in parks without (absence) or with (presence) gnawing hay blocks (significant interactions between group composition and presence/absence of gnawing blocks). Data are reported as the means ± standard deviations.

The presence of the hay blocks affected the use of the feeding and resting areas to different extents, depending on the group composition in parks (significant interaction absence/presence blocks × sex-group composition) (Figure 1). In parks without blocks, the time spent in the resting area was the highest in M parks; in contrast, in parks with blocks, this time increased in the rabbits in F parks to those of M and FM parks (Figure 1a). Similarly, the time spent resting in the resting area decreased in the rabbits in F parks to those of M and FM parks when blocks were not included, while an opposite trend was recorded in the presence of the blocks (Figure 1b). In contrast, while time spent resting in the feeding area (Figure 1c) and time spent resting with the stretched body (Figure 1d) did not change with sex-group composition in parks with blocks, large differences between F parks and FM parks were observed in the absence of blocks.

As age increased from 42 to 70 days, rabbits spent less time feeding and drinking (−35.2% and −14.3%, respectively; 0.01 < *p* < 0.001) and increased their time for caecotrophy (*p* < 0.001), self- and allo-grooming (+4.3% and +0.33 percentage points, respectively; *p* < 0.001), and biting/licking (+0.13 percentage points; *p* < 0.05) (Table 2). The time spent sitting and moving decreased with increasing age. Time resting with the crouched body decreased (−19.4%), whereas time resting with the stretched body increased (+41.4%) (*p* < 0.001) as age increased. 

**Figure 2:**
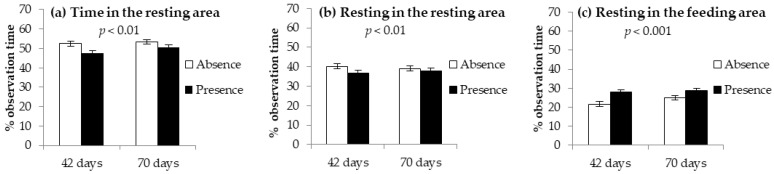
Time (% of observation time) spent (**a**) in the resting area, (**b**) resting in the resting area, (**c**) and resting in the feeding area of the parks by growing rabbits kept in collective parks without (absence) or with (presence) gnawing hay blocks at 42 and 70 days of age (significant interactions age × absence/presence of gnawing blocks). Data are reported as means ± standard deviations.

Significant interactions were recorded between age and the absence or presence of gnawing blocks for the use of the resting area (Figure 2). In fact, the time the rabbits spent in the resting area was the lowest at 42 days when the blocks were in the parks (Figure 2a), which coincided with the lowest time spent in the resting area (Figure 2b). In contrast, the lowest resting time in the feeding area was observed at 42 days for rabbits kept in parks without blocks (Figure 2c).

**Figure 3:**
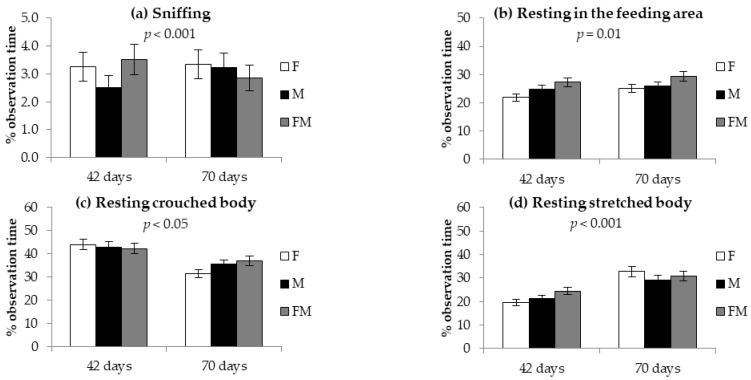
Time (% of observation time) spent (**a**) sniffing, (**b**) resting in the feeding area of the park, (**c**) resting with crouched body, and (**d**) resting with stretched body in growing rabbits kept in collective parks with only females (F), only males (M) or with males and females (FM) at 42 and 70 days of age (significant interactions between sex-group composition and age). Data are reported as means ± standard deviations.

As for sniffing and resting behaviors, rabbits in collective parks with different sex-group compositions showed differences according to age (significant interaction age × group composition; Figure 3). The lowest time spent sniffing at 42 days of age was recorded in parks with only males, whereas at 70 days of age, the lowest time was recorded in parks with both sexes (Figure 3a). The lowest time spent resting in the feeding area was recorded at 42 days in parks with only females, whereas the highest value was recorded in parks with mixed sexes at 42 and 70 days (Figure 3b). While no difference was observed in the time spent resting with the crouched body according to group composition at 42 days, at 70 days the lowest value was recorded for rabbits in F parks (Figure 3c). Finally, the time spent resting with the stretched body was lowest in single-sex parks at 42 days and highest in F parks at 70 days of age (Figure 3d).

### 3.3. Reactivity Tests

The rabbits that were provided with gnawing blocks spent more time moving during the open-field test (+9.9%; *p* < 0.05; Table 3) and approached the bottle earlier during the novel-object test (61.6% vs. 49.3%; *p* < 0.05; Table 4) compared to rabbits that were kept without blocks. Nevertheless, after five minutes of testing, no difference in the novel-object test or human-approach test was recorded between the rabbits of the two treatments (Table 4).

As for sex-group composition, during the open-field test, rabbits in parks with both sexes spent more time moving (+22.5% vs. rabbits of F and M parks; *p* < 0.05), while rabbits in parks with only males displayed self-grooming for a longer time (+49.2% vs. rabbits of F and FM parks; *p* < 0.01) (Table 3). In the human-approach test, some differences were recorded during the last minutes of the test; however, there were no marked differences in reactivity towards man among rabbits kept in parks with different sex-group compositions (Table 4).

## 4. Discussion

Under the conditions of conventional rearing systems, growing rabbits have traditionally been kept in barren enclosures with little space for movement and few possibilities of fully expressing their species-specific behaviors, such as gnawing and social interactions. Many of the behavioral needs of rabbits under farming conditions are not known, nor have the effects of their restrictions been assessed [1]. Thus, the present study was intended to contribute to improving knowledge about rabbits’ behavioral needs and to the development and optimization of alternative cage-free housing systems for rabbits.

### 4.1. Effect of Gnawing Blocks

Among other behaviors, whether it is recognized that rabbits under wild conditions have the opportunity of gnawing besides foraging, the presence of gnawing objects under farming conditions has been claimed to improve their welfare by increasing locomotion [22,23], reducing stress [8], and reducing negative social interactions in collective housing systems [6]. On the other hand, based on available literature, most studies reported that the provision of gnawing materials does not affect the productive performance of individually or park-housed growing rabbits [5,9,23], which was confirmed under the conditions of the present study.

The preference and time spent interacting with different gnawing objects can vary according to their physical and nutritional characteristics [5,9]. Wooden boards [8], cardboard and rubber chewing materials [24,25], straw [22], and gnawing blocks [23,25] have been tested. When comparing wooden sticks, soft-wood sticks were preferred over hard-wood sticks [5,9]. Indeed, the compressed hay blocks used in the present trial were expected to be rather soft to gnaw, and rabbits were found to have an average observation time of 0.4% (sniffing, licking, or gnawing). We hypothesize that interest in such objects could have been higher in the case of individuals than in collective housing. Nevertheless, even growing rabbits kept in individual cages spent little time (0.01–0.21% observation time) gnawing wooden sticks [26]. If considered simply as enrichments, one could argue that the low interaction time with the gnawing blocks depended on the reduction of the novelty effect over time [27] because they were continuously available. The position of the objects in the enclosures (on the floor, rather than on the ceiling or on a wall) could also modify their use, despite the implications for feces and bacterial contamination [28].

Under our conditions, despite the scarce effects on the budget time and the little time in absolute value addressed to the gnawing blocks, their presence affected the use of the different areas of the park and the animal distributions, in addition to animal reactivity towards a new environment in the open-field test and in the novel-object test that are worthy of consideration.

As for the use of the different areas of the park, in the present trial, rabbits spent 51.5% observation time in the resting area; they rested for 39.0% of observation time in the resting area and spent 26.7% observation time in the feeding area (averages of the two observation days). Morisse et al. [29] also showed that rabbits prefer to rest in the area of the park away from the feeders, likely because of less disturbance due to the animals’ access to feeders. Based on this preference, we also provided the resting area with a plastic floor, which has been proven to be preferred over a wire net by rabbits [30,31,32]. Nevertheless, the presence of blocks in the feeding area contributed to the presence of rabbits in this area compared to parks without blocks, which was likely due to the animals’ interest in this provision. In fact, when they were free to move among the four cages through swing doors, growing rabbits preferred cages provided with gnawing sticks [6]. In our study, this effect was confirmed over time (both at 42 and 70 days) and with different sex-group compositions.

Regarding behavior, previous studies performed on rabbits kept in cages in small groups (2–4 or 6) reported increased allo-grooming and social interactions [9,33] and decreased self-grooming with the provision of gnawing sticks, which was attributed to higher pheromonal olfactory stimulation and mutual recognition [33]. In contrast, in the parks used in the present study, in which social interactions were obviously higher than in cages, allo-grooming decreased in the parks with gnawing blocks. Gnawing sticks, as well as other environmental enrichments, have been found to reduce the incidence of abnormal behaviors in cages (e.g., stereotypies with cage manipulation) [34,35] and aggression among animals in collective systems [5,6]. In cages with six rabbits, Bozicovich et al. [9] reported increased aggressive behavior, but a lower occurrence of injured rabbits in enriched cages than in nonenriched ones. Under the conditions of our study, no evidence of this mitigation effect was found because of limited aggression and the absence of injured animals at the end of the trial, regardless of the treatment. The relatively early age at slaughter (73 days) and moderate group size (16 animals per group) could have accounted for this good result. In fact, aggressive interactions increase as animals approach sexual maturity, and injury occurrence has been reported in rabbits slaughtered later (15.0% and 22.0% at 76 and 83 days) [13] or kept in larger groups (15.6% at 68 days and 18.0% in groups with 27 and 36 rabbits at 75 days) [16] than in the present trial.

Finally, the results from the reactivity tests performed in the current study also indicate a positive effect of the provision of gnawing blocks, where a higher movement activity during the open-field test and a quicker approach to the novel object during the test can be considered as active and positive reactions to new stimuli [13,16,36]. On the other hand, the absence of differences in reactivity in the human approach test confirms previous observations about the positive effect of collective housing/conspecific presence in reducing fear levels towards men compared to rabbits housed individually. In fact, no difference in the reaction towards humans in the tonic immobility test was reported, even for rabbits kept in bicellular cages compared to those kept in small [36] or large groups [16,37].

### 4.2. Effect of Sex-Group Composition and Interaction among Experimental Factors

In view of growth performance, the present study confirmed that there is no need for single-sex rearing of growing rabbits, despite some differences in growth performance [12,13,14] or slaughter yield [13,38,39].

In contrast, under our conditions, the budget time varied according to group composition. In particular, increased resting time, and to a lesser extent, social interactions (allo-grooming) and decreased self-grooming, were observed in mixed-sex groups compared to single-sex groups. Similarly, a higher occurrence of social interactions and a lower occurrence of stereotypes were recorded by Bozicovich et al. [9] in rabbits kept in mixed-sex groups in cages of six compared to single-sex groups, whereas other authors did not report differences in behavior according to sex-group composition in cages [40]. Whether the above-mentioned differences in behavior can be interpreted as improved welfare for rabbits reared in mixed-sex groups is not definitively stated based on observations of injured animals. In fact, a higher rate of wounds was found in mixed-sex parks than in single-sex parks by Di Meo et al. [12] and in only male or mixed-sex parks than in only female parks [14] or cages [9]. In addition, in mixed-sex parks, the rate of injured rabbits was noticeably higher among males than females (25.8% vs. 11.3%; *p* ≤ 0.001) at the end of the fattening period [13]. Thus, aggression in mixed-sex parks is likely due to males, which is consistent with evidence that aggression is more severe among males than females, especially when sexual maturity approaches and rabbits are reared in large groups [11,41].

In accordance with other studies performed under different housing conditions [8,16,42,43], on average of the two observation days, our rabbits spent most of their time resting, self-grooming, feeding, sniffing, and drinking. As for the differences between 40 and 72 days of age, the greatest changes were due to the reduction in feeding time, as observed by other authors [16,28,36,44], and the increase in self-grooming. Differently, in larger parks (1.68 m^2^ vs. 1.00 m^2^ of the present trial) and with larger group size (20 and 27 animals per park), as age increased from 56 days to slaughtering age (76 and 83 days of age), the greatest change was the increased resting time, especially with a crouched body, at the expense of comfort behaviors (self- and allo-grooming) and exploration (sniffing, moving, running). Differences in the architecture of the park systems in the two studies (unique free area in Trocino et al. [16] and two connected modules in the present study) could also account for the different uses of space and time.

The use of the areas changed in parks with different sex-group compositions depending on the absence/presence of gnawing blocks, even if it is difficult to understand the reasons and welfare consequences for the observed differences.

As for the time the animals spent in the resting and feeding areas, the interactions we observed between rabbit age and the provision of gnawing blocks were likely related to the increase in animal size. On average, the rabbits were homogenously distributed between the resting (50% total observation time) and feeding areas (50%). At both ages, however, the time spent in the resting area was lower in parks with gnawing sticks than in parks without, but differences decreased with age (from −5.0 percentage units at 42 days to −2.9 percentage units at 70 days), likely because of the increased stocking density (kg live weight/m^2^) and the need for a comfortable spatial distribution in the two areas of the park.

## 5. Conclusions

With regard to knowledge about the rabbits’ behavioral needs, the changes in the rabbits’ distribution in the park proved the interest of the animals towards the provision of the blocks, which is consistent with the satisfaction of the animal’s behavioral needs. Although the rabbits spent a rather short time interacting with the compressed blocks used in the present study and showed few changes in the overall budget time, they showed an active and positive response towards a new environment and a new object when provided with the gnawing blocks, which can stand for reduced stress and improved welfare. Thus, the overall results related to production, behavior, and reactivity represent the provision of gnawing blocks in collective parks for welfare improvement, while they do not support the change from current mixed-sex rearing to single-sex. Finally, under the conditions of our study, no conclusion can be drawn regarding the effects on aggressive interactions, as no problem was recorded in this regard.

## Figures and Tables

**Table 1:** Effect of the presence of gnawing hay blocks and sex-group composition in collective parks on performance (31 to 72 days of age) and slaughtering results (73 days of age) of growing rabbits.

	Gnawing Hay Blocks (G)		Sex-Group Composition (S)			*p*-Value ^1^		RMSE ^2^
	Absence	Presence	Females	Males	Females + Males	G	S	
Parks (n)	9	9	6	6	6			
Rabbits (n)	140	136	91	93	92			
Live weight (g)								
at 31 days	765	767	768	766	764			82
at 52 days	1936	1979	1962	1952	1958			189
at 72 days	2845	2896	2880	2850	2881			220
Live weight gain (g/d)								
31 to 52 days	55.5	57.5	56.6	56.3	56.6	*		7.6
52 to 72 days	45.5	45.9	45.9	44.9	46.2			5.9
31 to 72 days	50.6	51.8	51.4	50.7	51.5	*		4.6
Feed intake ^3^ (g/d)								
31 to 52 days	118	121	119	120	119			4
52 to 72 days	165	169	168	166	168			7
31 to 72 days	141	145	143	143	144			5
Feed conversion ratio ^3^ (g/g)								
31 to 52 days	2.23	2.20	2.20	2.24	2.20			0.08
52 to 72 days	3.64	3.68	3.65	3.69	3.64			0.12
31 to 72 days	2.87	2.87	2.86 ^a^	2.89 ^b^	2.86 ^a^		*	0.02
Slaughter weight (g)	2763	2818	2808	2769	2792	*		212
Carcass weight (g)	1696	1730	1712	1713	1714			147
Slaughter yield (%)	61.2	61.3	60.8 ^a^	61.7 ^b^	61.3 ^ab^		**	1.7

^1^ Interactions between the main factors were not significant and have not been reported in table. ^2^ Root-mean-square error. ^3^ At a park level. * *p* < 0.05, ** *p* < 0.01. Means with different superscript letters in the same row differ significantly (*p* < 0.05; Bonferroni test).

**Table 2:** Effect of the presence of gnawing hay blocks, sex-group composition, and age on behaviors (% of observation time; number of events per park per observation period) of growing rabbits kept in collective parks across 24 h. Data are reported as means ± standard deviations.

	Gnawing Hay Blocks (G)		Sex-Group Composition (S)			Age (A)		*p*-Value ^1^		
	Absence	Presence	Females	Males	Females + Males	42 d	70 d	G	S	A
Parks (n)	9	9	6	6	6	18	18			
Rabbits (n)	140	136	91	93	92	276	276			
Feeding (%)	11.8 ± 8.0	11.2 ± 8.3	11.8 ± 8.3	11.8 ± 8.4	11.2 ± 7.8	14.1 ± 8.7	9.1 ± 6.7			***
Drinking (%)	1.74 ± 2.26	1.51 ± 2.18	1.59 ± 2.04	1.71 ± 2.44	1.57 ± 2.17	1.75 ± 2.30	1.50 ± 2.14			**
Caecotrophy (%)	0.25 ± 0.97	0.22 ± 0.88	0.15 ± 0.61	0.20 ± 0.90	0.35 ± 1.18	0.10 ± 0.57	0.37 ± 1.16			***
Sitting (%)	0.38 ± 0.72	0.26 ± 0.52	0.30 ± 0.53	0.32 ± 0.66	0.37 ± 0.69	0.42 ± 0.77	0.24 ± 0.44			***
Moving (%)	0.76 ± 0.75	0.68 ± 0.69	0.66 ± 0.61	0.84 ± 0.91	0.65 ± 0.59	0.90 ± 0.75	0.53 ± 0.64			***
Running (%)	0.16 ± 0.46	0.17 ± 0.46	0.16 ± 0.40	0.18 ± 0.51	0.16 ± 0.46	0.19 ± 0.48	0.14 ± 0.44			
Self-grooming (%)	14.8 ± 7.7	14.5 ± 7.8	15.4 ^b^ ± 7.6	15.8 ^b^ ± 7.9	12.8 ^a^ ± 7.4	12.5 ± 6.3	16.8 ± 8.3		***	***
Allo-grooming (%)	0.75 ± 1.50	0.44 ± 1.05	0.60 ^ab^ ± 1.25	0.46 ^a^ ± 1.07	0.73 ^b^ ± 1.53	0.43 ± 1.16	0.76 ± 1.41	***	**	***
Sniffing (%)	3.50 ± 4.32	3.08 ± 3.73	3.80 ± 5.00	2.92 ± 3.61	3.16 ± 3.25	3.29 ± 3.86	3.28 ± 4.21			
Biting/licking (%)	1.01 ± 2.19	0.78 ± 1.69	1.11 ± 2.27	0.82 ± 1.87	0.75 ± 1.68	0.83 ± 1.95	0.96 ± 1.97			*
Total resting (%)	64.8 ± 13.6	66.4 ± 12.8	64.2 ^a^ ± 13.7	64.7 ^a^ ± 13.0	67.8 ^b^ ± 12.6	65.2 ± 13.0	66.0 ± 13.4		**	
Resting crouched body (%)	38.7 ± 17.4	39.3 ± 15.8	37.9 ± 16.8	39.3 ± 16.8	39.8 ± 16.1	43.2 ± 16.6	34.8 ± 15.4			***
Resting stretched body (%)	26.0 ± 15.6	27.1 ± 14.5	26.3 ± 15.6	25.3 ± 15.0	28.0 ± 14.5	22.0 ± 13.2	31.1 ± 15.4			***
Time spent in the resting area (%)	53.5 ± 13.3	49.4 ± 11.9	52.3 ^b^ ± 13.7	52.0 ^ab^ ± 11.8	50.0 ^a^ ± 12.9	50.7 ± 16.1	52.1 ± 8.2	***	*	
Resting in the resting area (%)	40.2 ± 14.3	37.7 ± 12.7	39.1 ± 14.4	39.0 ± 12.9	38.7 ± 13.4	39.2 ± 16.1	38.7 ± 10.5	**		
Resting in the feeding area (%)	24.6 ± 13.0	28.7 ± 11.6	25.1 ^a^ ± 13.1	25.7 ^a^ ± 11.5	29.1 ^b^ ± 12.4	26.0 ± 14.1	27.2 ± 10.5	***	***	***
Contact with the enrichment ^2^ (%)	-	0.42 ± 1.58	0.13 ± 0.83	0.20 ± 1.07	0.30 ± 1.42	0.20 ± 1.01	0.22 ± 1.26	n.e.	n.e.	n.e.
Stretching (n)	0.19 ± 0.47	0.22 ± 0.47	0.19 ^a^ ± 0.43	0.17 ^a^ ± 0.44	0.26 ^b^ ± 0.53	0.23 ± 0.49	0.19 ± 0.45		*	
Hops (n)	0.07 ± 0.36	0.03 ± 0.18	0.03 ± 0.19	0.07 ± 0.37	0.05 ± 0.25	0.04 ± 0.28	0.06 ± 0.28	n.e.	n.e.	n.e.
Rearing (n)	1.23 ± 12.3	0.31 ± 1.19	1.33 ± 14.4	0.34 ± 0.76	0.65 ± 4.65	0.34 ± 0.82	1.20 ± 12.4	n.e.	n.e.	n.e.
Aggressive behaviors (n)	0.03 ± 0.28	0.03 ± 0.20	0.00 ± 0.06	0.02 ± 0.17	0.06 ± 0.38	0.00 ± 0.00	0.06 ± 0.34	n.e.	n.e.	n.e.

^1^ With the exceptions reported in Figure 1, Figure 2 and Figure 3, the interactions among the main factors were not significant and are not reported in table. ^2^ Time spent sniffing, licking, or biting the compressed hay block. * *p* < 0.05, ** *p* < 0.01, *** *p* < 0.001. n.e. = not estimable. Means with different superscript letters in the same row differ significantly (*p* < 0.05; Bonferroni test).

**Table 3:** Effect of the presence of gnawing hay blocks and sex-group composition age on behaviors of growing rabbits kept in collective parks during the open-field test at 64 days of age. Data are reported as the means ± standard deviations.

	Gnawing Hay Blocks (G)		Sex-Group Composition (S)			*p*-Value ^1^	
	Absence	Presence	Females	Males	Females + Males	G	S
Parks (n)	9	9	6	6	6		
Rabbits (n)	54	54	36	36	36		
Entered animals (%)	59.3 ± 56.6	57.4 ± 60.3	52.8 ± 61.2	61.1 ± 53.4	61.1 ± 57.0		
Latency (s)	19.3 ± 12.2	26.4 ± 15.5	15.9 ± 11.0	26.4 ± 14.4	25.0 ± 15.2		
Total displacements (n)	32.6 ± 24.8	32.8 ± 25.3	30.2 ± 23.8	31.8 ± 24.1	36.1 ± 27.1		
Central displacements (s)	2.19 ± 3.22	1.63 ± 2.82	1.89 ± 3.23	1.39 ± 2.09	2.44 ± 3.56		
Exploration (s)	366 ± 67	365 ± 50	360 ± 53	368 ± 71	369 ± 53		
Movement (s)	29.4 ± 20.8	32.3 ± 22.9	28.5 ^a^ ± 21.1	28.8 ^a^ ± 18.9	35.1 ^b^ ± 25.0	*	*
Running (s)	2.20 ± 4.17	1.37 ± 3.89	1.56 ± 3.22	2.28 ± 5.32	1.53 ± 3.28		
Standing still (s)	55.6 ± 45.9	67.1 ± 52.6	70.1 ± 45.9	57.9 ± 50.6	56.1 ± 51.9		
Self-grooming (s)	3.28 ± 4.47	3.96 ± 6.36	2.94 ^a^ ± 4.49	4.64 ^b^ ± 7.08	3.28 ^a^ ± 4.47		**
Escape attempts (n)	0.16 ± 0.45	0.26 ± 0.77	0.05 ± 0.23	0.09 ± 0.29	0.45 ± 0.96	n.e.	n.e.
Resting (s)	17.5 ± 52.7	7.57 ± 24.5	13.4 ± 37.7	14.7 ± 50.3	9.53 ± 30.0	n.e.	n.e.
Biting (s)	5.54 ± 1.27	2.41 ± 6.66	2.92 ± 8.41	3.86 ± 10.22	5.14 ± 11.83	n.e.	n.e.
Digging, (s)	0.41 ± 2.34	0.15 ± 0.66	0.19 ± 0.75	0.11 ± 0.46	0.53 ± 2.84	n.e.	n.e.
Urination (n)	0.02 ± 0.14	0.00 ± 0.00	0.00 ± 0.00	0.00 ± 0.00	0.03 ± 0.17	n.e.	n.e.
Rearing (n)	1.24 ± 2.91	0.91 ± 2.54	1.11 ± 2.82	0.30 ± 0.75	1.80 ± 3.62	n.e.	n.e.
Hops (n)	0.02 ± 0.14	0.00 ± 0.00	0.00 ± 0.00	0.00 ± 0.00	0.03 ± 0.17	n.e.	n.e.

^1^ Interactions between the main factors were not significant and not reported in table. * *p* < 0.05, ** *p* < 0.01. n.e. = not estimable. Means with different superscript letters in the same row differ significantly (*p* < 0.05; Bonferroni test).

**Table 4:** Effect of the presence of gnawing hay blocks and sex-group composition on the rabbit–object contacts (rabbits that touched the object, % present rabbits) during the novel-object test at 65 days of age and on the rabbit–man contacts (rabbits that touched the man, % present rabbits) during the human-approach test at 67 days in growing rabbits kept in collective parks. Data are reported as the means ± standard deviations.

	Gnawing Hay Blocks (G)		Sex-Group Composition (S)			*p*-Value ^1^	
	Absence	Presence	Females	Males	Females + Males	G	S
Parks (n)	9	9	6	6	6		
Rabbits (n)	140	136	91	93	92		
							
Novel-object test							
Rabbit–object contacts (%)							
1–60 s	49.3 ± 49.7	61.6 ± 50.1	57.9 ± 49.6	62.1 ± 49.2	45.6 ± 50.8	*	
61–180 s	22.9 ± 42.6	13.0 ± 32.7	14.7 ± 36.2	16.8 ± 37.6	22.8 ± 45.1	*	
181–300 s	9.03 ± 74.6	5.08 ± 31.2	6.32 ± 21.9	6.32 ± 86.8	9.80 ± 30.7		
1–300 s	81.2 ± 39.0	80.4 ± 40.8	78.9 ± 41.0	85.3 ± 34.7	78.3 ± 42.2		

Human-approach test							
Rabbit–man contacts (%)							
1–60 s	30.6 ± 46.1	34.5 ± 49.0	35.8 ± 49.6	28.4 ± 45.3	33.3 ± 47.0		
61–180 s	8.3 ± 51.8	7.9 ± 71.1	3.2 ± 51.0	10.5 ± 30.9	10.7 ± 30.7		
181–300 s	6.3 ± 59.2	11.5 ± 77.0	3.2 ^a^ ± 48.0	13.7 ^b^ ± 34.7	9.7 ^b^ ± 27.8		*
1–300 s	45.1 ± 52.1	54.0 ± 51.3	42.1 ± 52.5	52.6 ± 50.1	53.7 ± 50.8		

^1^ Interactions between the main factors were not significant and are not reported in Table * *p* < 0.05. Means with different superscript letters in the same row differ significantly (*p* < 0.05; Bonferroni test).

## Data Availability

The datasets analyzed in the current study are available from the corresponding author upon reasonable request.
